# Crosstalk Between ER Stress, Autophagy and Inflammation

**DOI:** 10.3389/fmed.2021.758311

**Published:** 2021-11-05

**Authors:** Sandhya Chipurupalli, Unni Samavedam, Nirmal Robinson

**Affiliations:** ^1^Cellular-Stress and Immune Response Laboratory, Center for Cancer Biology, University of South Australia and SA Pathology, Adelaide, SA, Australia; ^2^College of Medicine, University of Cincinnati, Cincinnati, OH, United States

**Keywords:** autophagy, unfolded protein response, ER-stress, cytokines, inflammation

## Abstract

The endoplasmic reticulum (ER) is not only responsible for protein synthesis and folding but also plays a critical role in sensing cellular stress and maintaining cellular homeostasis. Upon sensing the accumulation of unfolded proteins due to perturbation in protein synthesis or folding, specific intracellular signaling pathways are activated, which are collectively termed as unfolded protein response (UPR). UPR expands the capacity of the protein folding machinery, decreases protein synthesis and enhances ER-associated protein degradation (ERAD) which degrades misfolded proteins through the proteasomes. More recent evidences suggest that UPR also amplifies cytokines-mediated inflammatory responses leading to pathogenesis of inflammatory diseases. UPR signaling also activates autophagy; a lysosome-dependent degradative pathwaythat has an extended capacity to degrade misfolded proteins and damaged ER. Thus, activation of autophagy limits inflammatory response and provides cyto-protection by attenuating ER-stress. Here we review the mechanisms that couple UPR, autophagy and cytokine-induced inflammation that can facilitate the development of novel therapeutic strategies to mitigate cellular stress and inflammation associated with various pathologies.

## Introduction

Inflammation is the primary defense mechanism mounted to protect the host against infection when cells sense pathogen associated molecular patterns (PAMPs) through the pattern recognition receptors (PRRs). Inflammation can also be triggered when the immune system senses substances released by damaged cells known as damage associated molecular patterns (DAMPs) which are also recognized by PRRs. Profound and chronic inflammation is damaging hence, associated with pathological conditions such as intestinal bowel disorder (IBD), chronic obstructive pulmonary disease (COPD), diabetes, obesity, cardiovascular diseases, multiple sclerosis and rheumatoid arthritis. On the other hand, there is epidemiological, clinical andexperimental evidence that inflammation can be triggered in the absence of PRR-mediated sensing and -signaling through cellular stress, wherein biological processes within cellsare impaired resulting in increased inflammation linked to the above-mentioned pathologies (1).

When cells are stressed, they trigger signaling pathways which enable the cells to adapt to the changes caused by the stress. For a cell, these perturbations can be either life-enhancing or life-threatening (2). Likewise, any chronic perturbations disturbing the endoplasmic reticulum (ER) homeostasis results in ERstress. ERstress is characterized by the accumulation of aberrant proteins which influence the protein folding capacity of the ER (3). The cell responds to ER stress by initiating unfolded protein response (UPR), which is aimed at increasing the ability of ER to fold proteins properly, regulate protein translation and induce cell death if everything fails. Interestingly, UPR proteins also regulate inflammation associated with diseases (4). Autophagy is another process which is activated upon cellular stress to reestablish homeostasis. It aids in the lysosomal degradation of damaged organelles, denatured proteins and pathogens. Autophagy also plays a critical role in regulating inflammation by promoting the immune cell-survival and regulating the expression and secretion of inflammatory cytokines (5). Intriguingly, the UPR pathways that regulate inflammation also intersect with mechanisms that regulate autophagy and together govern the outcome of inflammatory diseases. Therefore, in this review we describe the molecular links between UPR, autophagy and inflammation and their involvement in some of inflammatory diseases.

## ER Stress and UPR Mechanisms

The endoplasmic reticulum (ER) is responsible for major cellular functions such as protein folding, synthesis of lipids, sterols and calcium storage (2, 6). ER function can be influenced by a wide variety of factors. For instance, hypoxia, glucose deprivation, hyperthermia, acidosis, calcium levels, altered metabolism, infections, mutations in secretory proteins and inflammation can disturb appropriate functioning of the ER, impacting protein folding (7). This causes an imbalance between the demand for protein folding and the capacity of the ER for protein folding resulting in the accumulation of misfolded/unfolded proteins (2, 6, 7). This condition is termed as “ER stress” which has been implicated in various inflammatory conditions associated with cardiovascular, neurodegenerative and metabolic diseases (8).

In response to ER stress, unfolded protein response (UPR) is activated (9, 10). UPR comprises of three distinct signal transduction arms mediated through, protein kinase RNA (PKR)-like ER kinase (PERK), inositol-requiring protein-1 (IRE1) and activating transcription factor-6 (ATF6). Each UPR activator protein consists of three domains; ER luminal domain (LD), a single membrane-spanning domain, and a cytosolic domain (CD). Under physiological conditions, PERK, IRE1 and ATF6 are bound via their LD to binding immunoglobulin (BiP) and remain in inactive state (11, 12). BiP is the most abundant Hsp70-type ER chaperone and a direct ER stress sensor (12, 13). Upon accumulation of misfolded/unfolded proteins, BiP then initiates UPR by sequestering away from the luminal domains of PERK, IRE1 and ATF6 and binds to nascent polypeptidesto chaperone proper folding andattain native conformation (13). Dissociation of BiP from IRE1, PERK and ATF6activatesthe three distinct UPR branches (14). Subsequently, UPR regulates the rate of protein synthesis, translocation of proteins into the ER, chaperoning the misfolded proteins, andprotein trafficking (15). Thus,UPR signal transducers are critical for ER quality control and maintaining ER homeostasis.

## ER Stress Induced Autophagy

Increased presence of misfolded/unfolded protein load in the ER is harmful to cells and therefore cells have evolved mechanisms that can detect, unfold and refold the misfolded/unfolded proteins (6). However, the misfolded proteins that cannot be repaired are eliminated from the cell through the specialized processes, ER-associated protein degradation (ERAD) and autophagy (7, 15). ERAD involves recognition of aberrant proteins by the molecular chaperones and translocation of those damaged proteins from the ER back into the cytoplasm, where they are delivered to the proteasome for degradation (7, 16). However, ERAD has been extensively reviewed elsewhere and is not the prime focus of this review (16, 17). Moreover, excessive or sustained ER stress triggers apoptosis to remove the affected cell (18, 19). However, when these mechanisms remain unsuccessful to restore ER homeostasis or recover ER, other stress-response pathways such as autophagy (macroautophagy) may be initiated to selectively eliminate the misfolded/unfolded proteins and damaged ER.

Autophagy is the major lysosomal degradation pathway characterized by the sequestration of the damaged cytoplasmic components by double-membrane bounded vacuoles called autophagosomes (20, 21). This process has been extensively reviewed by others (22, 23) and therefore the fundamental steps in the formation and maturation of autophagosomes are not discussed here in detail. Nevertheless, the association between autophagy and ER stress is not yet fully understood. Therefore, in this review, we have summarized the current findings that integrate the signaling pathways linking autophagy and ER stress.

Each arm of the UPR regulates autophagy in different ways during ER stress. Upon ER-stress, IRE1dissociates from BiP and gets activated (11, 24), which then recruits tumor necrosis factor receptor-associated factor 2 (TRAF2)and apoptosis signal-regulating kinase-1 (ASK1) resulting in the activation of c-Jun N-terminal kinases (JNKs). Active JNK mediates the phosphorylation of Bcl-XL/Bcl-2, leading to the release of Beclin-1 (BECN1) and enhanced basal autophagy (11, 25). PERK arm of UPR also regulates autophagy through the activation of ATF4 and CHOP, which drives the expression of autophagy proteins, autophagy-related (ATG)-12 and ATG5, respectively. ATG12 and ATG5 complex with ATG16L to initiate the formation of autophagosomes (26). CHOP also activates tribbles-homolog3(TRIB3) which inhibits AKT/mechanistic-target-of-rapamycin (mTOR) signaling leading to the induction of autophagy (27). Additionally, calcium released from the ER activates enzymes such as death-associated protein kinase (DAPK), protein-kinase-Cθ (PKCθ) or AMP-activated protein kinase (AMPK), which positively regulate autophagy by inhibiting mTOR (21). However, these signaling pathways have been implicated under different stress conditions, thus they could be functioning in a context-dependent manner.

## ER Stress and Inflammation or Inflammatory Cytokine Regulation Underlies ER Stress

A well-regulated protein homeostasis is crucial for better execution of basic cellular functions. ER plays a significant role in folding and modifying the secretory as well as membrane proteins, thus maintaining proteostasis (28). Perturbations in protein homeostasis lead to ER stress, which activates UPR and inflammation even in the absence of infection (29). However, ER stress-induced inflammation associated with pathologies such as diabetes, obesity, atherosclerosis, and cancer has been shown to be detrimental. Thus, the destructive or protective nature of ER-stress regulated inflammation depends on the intensity, and type of immune response. Acute induction of ER stress and inflammation safeguard the cell viability and functions while chronic induction can be destructive. Thus, ER stress and ER stress-induced inflammation act in a context-dependent manner. ER stress-induced UPR signaling is coupled with the activation of pro-inflammatory pathways, mediated by nuclear factor kappa light chain enhancer of activated B cells (NF-κB) (4). All the three ER stress sensors; IRE1, PERK and ATF6 induce inflammation by activating NF-kB via different mechanisms leading to the transcription of genes that encode for inflammatory cytokines (29, 30). Under basal conditions, NF-κB forms a complex with inhibitor kappa B (IkB) which prevents the translocation of NF-κB and subsequent transcriptional function. Upon activation of PRR signaling, IκB is phosphorylated and degraded by proteasomes allowing NF-κB to translocate into nucleus and induce the expression of cytokines (31).

IRE1, the evolutionarily conserved signal transducer of the UPR plays a significant role in basic cellular functions and in various pathological conditions associated with inflammation (32). Upon activation of UPR, IRE1 is phosphorylated resulting in the recruitment of TRAF2 and ASK-1 which subsequently activates JNK and NF-κB, leading to the production of inflammatory cytokines. Additionally, IRE1/TRAF2/ASK1 complex activates inhibitory kappa B kinase (IKK), which phosphorylates IκB, allowing the translocation of NF-κB into the nucleus where cytokine gene expression is induced. PERK is also shown to activate NF-κB triggering persistent inflammatory response through the expression of genes that encode inflammatory cytokines like interleukin-1 (IL-1), interleukin-6 (IL-6), and tumor necrosis factor alpha (TNF-α). PERK also regulates NF-κB and apoptosis through the activation of eIF2a-ATF4-CHOP axis of UPR. Activation of PERK downregulates global protein translation, which preferentially affects IκB expression over NF-κB as IκB has a shorter half-life, enabling NF-κB to translocate. ATF6 also positively affects NF-κB activation via mTOR/AKT signaling. Furthermore, reactive oxygen species (ROS) generated because of calcium dysregulation during ER-stress could activate inflammation through NF-κB dependent or independent mechanisms.

Transcription factors such as activator-protein-1 (AP-1) regulated by mitogen activated protein kinases (MAPK) such as p38 work in concert with NF-κB to induce cytokines. Interestingly, IRE1, PERK and ATF6 have been reported to induce inflammation by promoting extracellular-signal-regulated kinase (ERK) and p38. UPR is also known to activate cytokines such as interferon-β (IFN-β) through interferon-responsive-factor-3 (IRF3). Although the precise mechanism remains elusive, stimulator-of-interferon-gene (STING) activation due to calcium disruption induces ER-stress (33, 34). Mitochondrial-DNA released as a result of ER-stress induced mitochondrial damage is also known to activate STING (35). As noted, certain types of ER stress mobilize STING translocation and STING-dependent IFN-I production (33).

**Figure 1:**
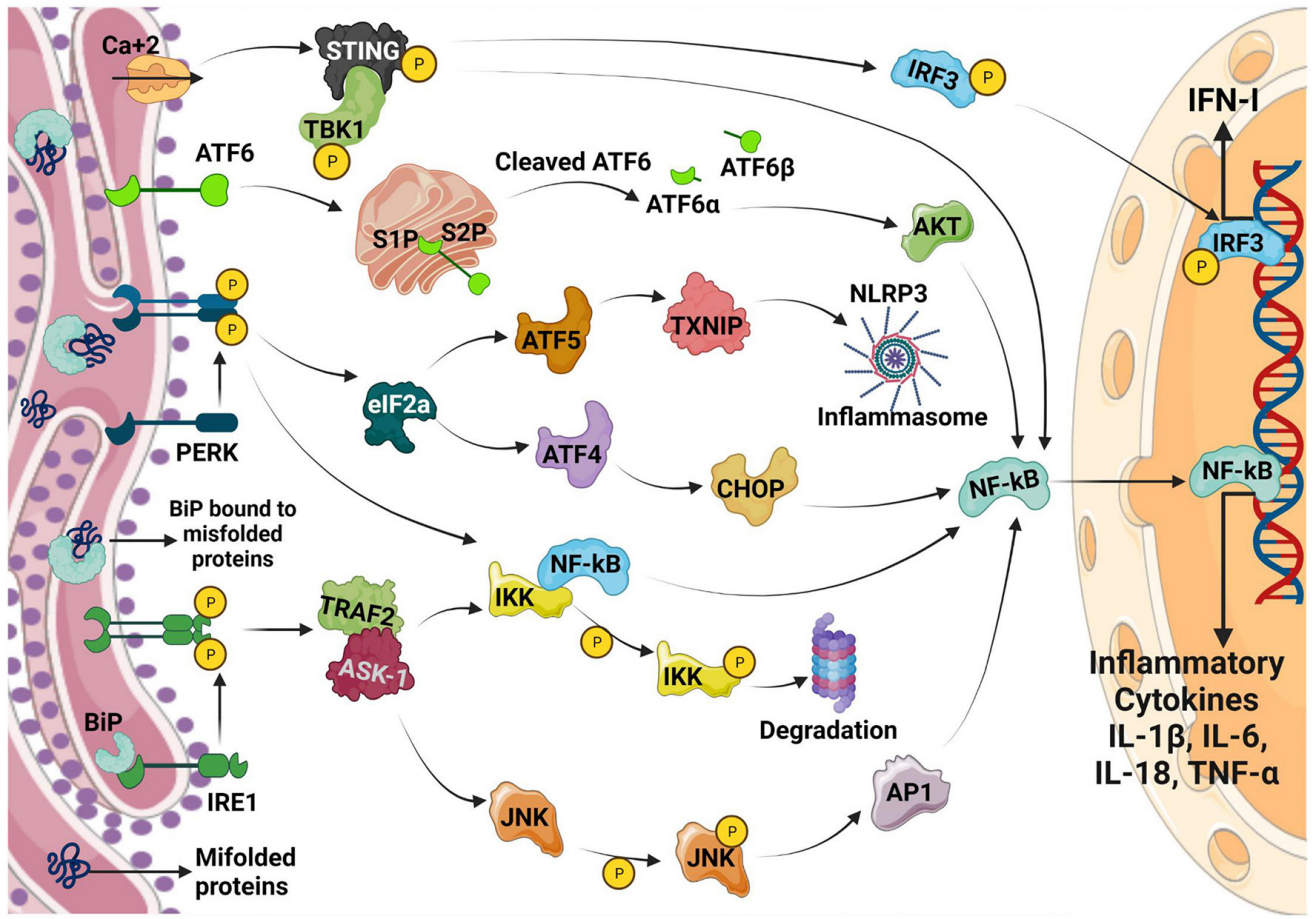
Inflammatory/Cytokine signaling regulated by UPR. Upon ER stress, the ER chaperone BiP dissociates from its complex with IRE1, PERK and ATF6 which results in the activation of the three arms of the UPR pathway as shown in the figure. All the three arms of the UPR regulates the production of inflammatory cytokines via different mechanisms which converge on NF-κB activation. PERK also regulates TXNIP through the induction of ATF5 which in turn modulates NLRP3 inflammasome resulting in enhanced inflammation. Calcium disruption induced ER stress activates STING pathway resulting in the production of IFN-1.

In addition to UPR activating inflammatory transcription factors NF-κB and IRF3, UPR arms directly regulate cytokine expression (Figure 1). Chromatin immunoprecipitation (ChIP) analyses have revealed the binding of XBP1 to the promoters of the IL-6, TNF-α and IFN-βencoding genes (36, 37). Similarly, ATF4 (38) and CHOP (39) bind to IL-6 and IL-23a promoters, respectively.

ER-stress pathways are also known to directly impact on pattern-recognition-receptors (PRRs). ER stress results in inflammasome activation and IL-1β production possibly resulting in pyroptosis. IRE1 and PERK are also known to upregulate thioredoxin-interacting-protein (TXNIP) (40–42) by abrogating micro-RNA (miR)-17. However, PERK directly increases TXNIP expression through ATF5 (41). TXNIP regulates NLRP3 inflammasome, a multicomponent complex that contains caspase-1 and induces the caspase-1–dependent secretion of the pro-inflammatory cytokines IL-1β and IL-18. TXNIP dependent inflammasome activation occurs on mitochondria resulting in mitochondrial damage and further increase in inflammation. IRE1 is also shown to stimulate nucleotide-binding oligomerization domain-containing protein 1/2 (NOD1/2)-mediated production of inflammatory cytokine IL-6 during *Brucella abortus* infection (43). IRE1a also contributes to the lipid-induced activation of NLR family pyrin domain containing 3 (NLRP3) inflammasome (32). This could be inhibited using tauroursodeoxycholic acid (TUDCA) and the IRE1 kinase inhibitor, KIRA6. Furthermore, IRE1 regulates IL-1β and IL-18 expression through the activation of glycogen-synthase-kinase-3β (GSK3β). ER stress may also enable cells to produce IL-1β in response to TLR4 ligation in a TRIF (TIR domain containing adaptor protein inducing interferon beta)-dependent and caspase 8-dependent, but XBP1 and CHOP independent manner (44). Although PRR and ER-stress can induce inflammation directly, PRR stimulation after ER-stress synergistically induces profound inflammation which has been demonstrated using pharmacologic UPR inducers and XBP1 over-expression (36, 39, 45, 46).

On the other hand, PERK, another ER stress sensor is also shown to activate the downstream signaling pathways leading to the dissociation of NF-κB from IkB and its subsequent translocation into the nucleus. This triggers persistent inflammatory responses by activating the expression of genes that encode inflammatory cytokines like IL-1, IL-6, and TNF-α. Additionally, PERK activates its downstream signaling eIF2a-ATF4-CHOP pathway and NF-κB which initiates inflammation and apoptosis. ERS leads to dissociation of TRAF from TRAF2-procaspase 12 complex, which is located on the ER membrane, leading to activation of caspase 12. The ATF6 pathway also activates NF-κB, further intensifying the expression of inflammatory genes, which secrete more cytokines.

## Crosstalk Between ER Stress-Autophagy-Inflammation in Disease Progression

**Figure 2:**
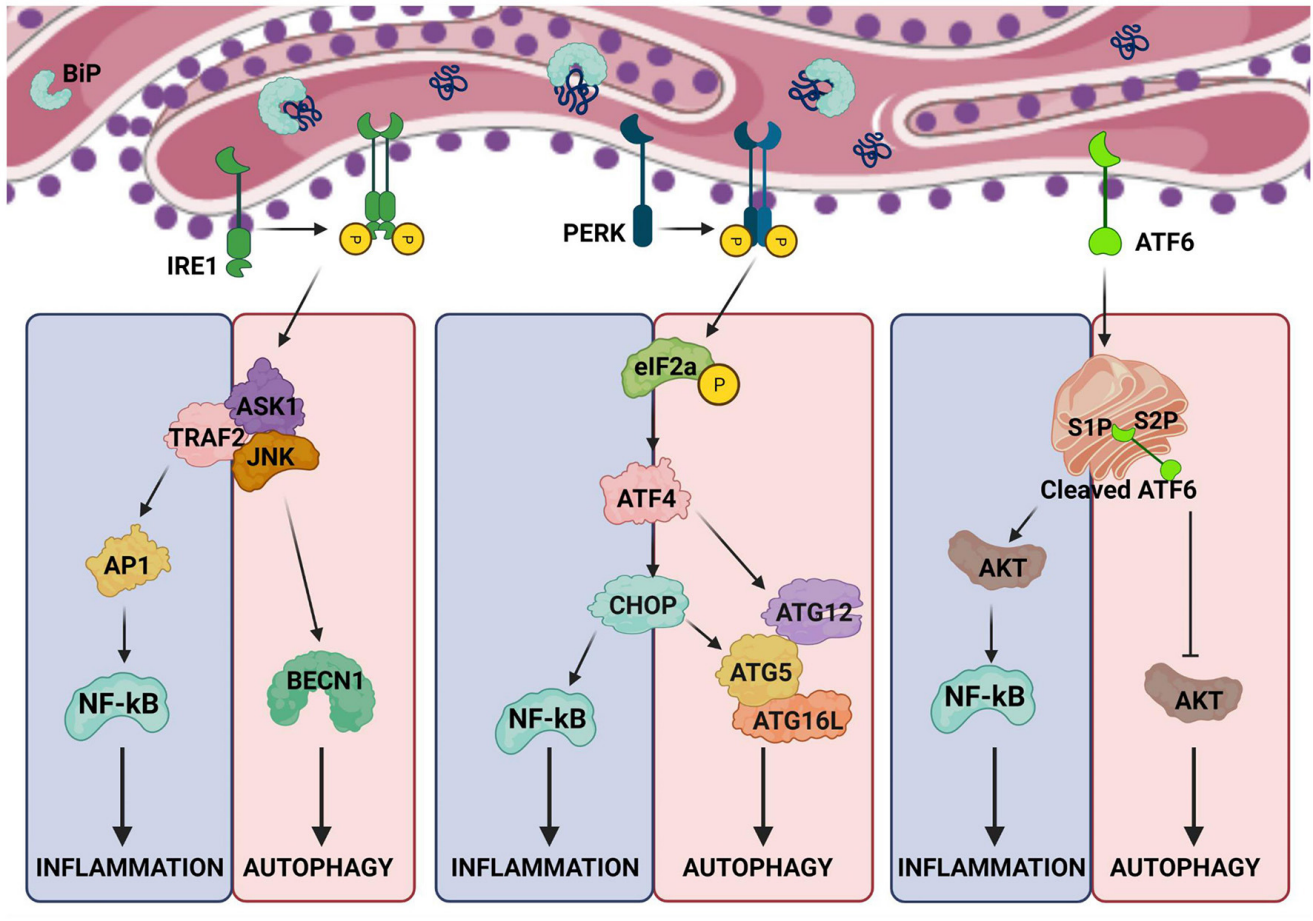
Crosstalk between ER stress, autophagy and inflammation. The arms of UPR activating inflammation also intersect with pathways regulating autophagy. The possible points of intersection are shown in the illustration.

PRR induced-inflammation is beneficial in mounting an immune response against microbes. However, profound inflammation in the absence of infection is pathologic which is supported by the clinical use of antibodies against inflammatory cytokines to treat diseases such as intestinal bowel disease, rheumatoid arthritis, and multiple sclerosis.As discussed above, UPR signaling and autophagy are intertwined with inflammation (Figure 2). Therefore, UPR has been linked with several inflammatory diseases and some of which has been reviewed here below.

### Intestinal Bowel Disease

Intestinal epithelial cells (IECs) are constantly exposed to microbiota, metabolites and toxins which force them to produce large amounts of cytokines and various other proteins resulting in ER-stress. Although, UPR helps in resolving ER-stress, continued ER-stress and disruptions in the UPR mechanism can result in chronic inflammation. Therefore, it is no surprise that studies have associated UPR dysregulation with Crohn's disease (CD) and ulcerativecolitis (UC), two major types of IBDs (47, 48). IBD is also one of the first polygenic disease to be genetically linked to UPR components (47). Intestinal inflammation is primarily linked to IRE1-XBP1 arm of the UPR pathway because mice deleted of the IRE1 gene in mouse intestinal epithelium are more susceptible to dextran sulfate sodium (DSS)-induced colitis (49). Similarly, mice deficient in XBP-1 in the intestine develop spontaneous intestinal inflammation and immune infiltration resembling IBD (50).

The barrier between microbial flora of the gut and IECs is maintained by the secretion of mucin2 (*MUC2)*. “Winnie” and “Eeyore”mice engineered with a single nucleotide polymorphism (SNP) expresses misfolded MUC2 resulting in strong UPR induction, and gut inflammation (51, 52). UPR and intestinal inflammation has been also linked in humans. For instance, anterior gradient 2 (AGR2) encoding a protein-disulfide-isomerase which enables protein folding and orosomucoid-like 3 (ORMDL3), which regulates ER calcium have been shown to induce UPR (53–55). Interestingly, Agr2^−/−^ mice develop severe ileo-colitis which is associated with misfolded mucin induced-ER stress (56). Furthermore, genome-wide association studies (GWAS) have mapped the XBP-1 gene locus as an IBD susceptibility region (57, 58). As described before, UPR interacts with autophagy pathways at multiple levels. UPR induces autophagy and reciprocally, autophagy may limit UPR by reducing ER-stress (59). Interestingly, a core autophagy effector protein ATG16L is associated with IBD in humans. Consistently, mice deficient in ATG16L1 in IECs develop Crohn's like disease (60–62). Furthermore, deletion of ATG16L1 and XBP1 in IECs results in more severe IBD suggesting that autophagy and UPR synergizes in regulating intestinal inflammation (61).

### Chronic Obstructive Pulmonary Disease

UPR is also associated with the pathogenesis of chronic obstructive pulmonary disease (COPD). External stimulants such as cigarette smoke induces ROS production which disturbs the redox environment thus preventing proper protein folding by modulating the protein-disulfide-isomerase (PDI) (63). Dysregulation of protein folding in lung and bronchial epithelial cells induces UPR (64, 65). Furthermore, oxidative damage of proteins caused by cigarette smoke leads to impaired degradation of misfolded, non-functional proteins triggering UPR (66). Cigarette smoke induced-UPR is characterized by PERK-eIF2a-mediated CHOP induction (64, 66–68). On the other hand, cigarette smoke also activates ERK1/2 and NF-kB regulated inflammation (69, 70). Impaired autophagy has been linked to cigarette smoke induced inflammation. On the contrary, activating autophagy using mTOR inhibitor rapamycin results in increased apoptosis and inflammation (71). Interestingly, another form of autophagy known as chaperone-mediated autophagy (CMA), which is LAMP2A facilitated selective degradation of proteins containing Lys-Phe-Gln-Arg-Gln (KFERQ) in the lysosomes mitigates cigarette smoke induced UPR and apoptosis (72).

### Neurodegenerative Disorders

#### Multiple Sclerosis

MS is an autoimmune disorder in which the T-cells target myelin sheath (73). ER-stress induced UPR is found to be a hallmark of MS (74). It is proposed that autophagy-induced cell death could be a possible mechanism by which UPR resolves ER-stress. Hence, autophagy is elevated in MS-lesions resulting in demyelination and neuro-inflammation. PERK and CHOP activation has been found to be consistent with upregulation of BAX and BCL2 in experimental autoimmune encephalomyelitis (EAE). However, the molecular mechanisms integrating UPR, autophagy and inflammation has not been completely understood (3).

#### Parkinson's Disease

Parkinson's Disease (PD) is a neurodegenerative disease and numerous evidences suggest that inflammation exacerbates the disease (75). Reports have also linked the role of ER-stress in the pathogenesis of PD using neurotoxic models of PD. Interestingly, depletion of CHOP protects dopaminergic neurons against hydroxydopamine (6-OHDA) indicating the involvement of ER-stress in PD (76). Similarly, silencing XBP1 another UPR arm results in chronic ER stress and dopaminergic neuron degeneration (77). Parkin an E3 ubiquitin ligase implicated in Parkinson's disease is a key regulator of mitochondria-specific autophagy (mitophagy). Interestingly, ATF4 upregulates parkin by directly binding to the promoter region upon ER stress (78). Although, studies addressing the cross talk between ER stress, autophagy and inflammation in PD are limited, UPR can co-regulate inflammation and autophagy as discussed above.

#### Cardiovascular Diseases

Inflammation and autophagy are known to play a key role in the progression of cardiovascular diseases (CVDs) such as atherosclerosis, ischemia and/or reperfusion. Intriguingly, ER stress that is implicated in inflammation and autophagy has been currently coupled with the pathophysiological aspects of the cardiovascular system (CVS) (79). Upregulation of UPR is observed in cardiac hypertrophy and heart failure. Inflammation and ER stress within the CVS are connected through various regulators such as NF-κB, JNK, spliced XBP-1 and ROS (80–82). As discussed in earlier sections of this review, UPR activation leads to recruitment of TRAF2 by IRE1 which interacts with JNK and IκB resulting in the activation of downstream inflammatory signaling and cytokine production. Additionally, IRE1 auto-phosphorylates and splices its downstream XBP-1 which stimulates the production of inflammatory cytokines by enhancing Toll-like receptor (TLR) signaling (32, 83). ATF6 activation also results in transcriptional activation of inflammatory proteins like C-reactive protein (CRP) which fosters the expression of monocyte chemoattractant protein-1 (MCP-1) and contributes to inflammation (84, 85). Furthermore, ATF6 phosphorylates AKT and activates NF-κB which stimulates the expression of various cytokines (86). Similarly, PERK also triggers NF-κB-induced cytokine signaling by activating IκB (87).

It is well-established that ER stress is also implicated in atherosclerosis where UPR activation is observed in macrophage-derived and smooth muscle cell (SMC)-derived foam cells (79, 88). The plaque deposition in the arterial walls triggers infiltration of macrophages and neutrophils leading to production of IL-1 and IL-6 (89). Additionally, ROS is induced resulting in UPR activation which can further enhance inflammation and tissue damage (89). Inflammation induced mitochondrial damage and ROS can in turn induce autophagy (90). A weak association between autophagy and plaque formation has been reported based on the expression of autophagy markers (91, 92). However, whether autophagy is beneficial or detrimental in atherosclerosis is poorly understood.

## Conclusion

Traditionally, engagement of PRRs with PAMPs has been considered the primary trigger for inflammation. However, changes in intracellular functions causing cellular stress have been lately recognized to play a key role in inflammation associated with pathologies. Thus, ER stress-induced inflammation has been implicated in several inflammatory diseases. Although response to ER-stress (UPR) aids in mitigating ER-stress, UPR pathways also promote inflammation and diseases such as diabetes, obesity, IBD, inflammatory lung disorders, cardiovascular diseases and cancer. Moreover, UPR pathways are interlinked with other cellular-stress response mechanisms such as autophagy which can potentially mitigate inflammation and disease progression. Conversely, activation of cellular homeostasis mechanisms such as autophagy can be an impediment to treat diseases such as cancer. However, UPR induced inflammation and autophagy vary between diseases and is cell type dependent. Although, inflammation and autophagy have been reported during ER-stress, it is correlative. Therefore, molecular mechanisms that integrate UPR, autophagy and inflammation need to be elucidated which is crucial for therapeutic targeting.

## Author Contributions

SC: prepared the manuscript draft and figures. All authors contributed to the article and approved the submitted version.

## Funding

Research in the laboratory of NR was supported by funds from the Center for Cancer Biology, University of South Australia and Neurosurgical Research Foundation, Adelaide, Australia.

## Conflict of Interest

The authors declare that the research was conducted in the absence of any commercial or financial relationships that could be construed as a potential conflict of interest.

## Publisher's Note

All claims expressed in this article are solely those of the authors and do not necessarily represent those of their affiliated organizations, or those of the publisher, the editors and the reviewers. Any product that may be evaluated in this article, or claim that may be made by its manufacturer, is not guaranteed or endorsed by the publisher.
